# The proofreading mechanism of the human leading strand DNA polymerase ε holoenzyme

**DOI:** 10.1101/2025.04.11.648458

**Published:** 2025-04-12

**Authors:** Feng Wang, Qing He, Michael E O’Donnell, Huilin Li

**Affiliations:** 1 Department of Structural Biology, Van Andel Institute, Grand Rapids, MI, USA; 2 DNA Replication Laboratory and Howard Hughes Medical Institute, The Rockefeller University, New York, NY, USA

**Keywords:** DNA proofreading, DNA Polymerase epsilon, PCNA, exonuclease proofreading, leading strand DNA, leading strand fidelity

## Abstract

The eukaryotic leading strand DNA polymerase epsilon is a dual function enzyme with a proofreading exonuclease site located 40 angstroms from the DNA synthesizing polymerase site. Errors in Pol epsilon proofreading can cause various mutations, including C to G transversions, the most prevalent mutation in cancers and genetic diseases. Pol epsilon interacts with all three subunits of the PCNA ring to assemble a functional holoenzyme. Despite previous studies on proofreading of several polymerases, how Pol epsilon, or any Pol complexed with its sliding clamp proofreads a mismatch generated in situ has been unknown. We show here by cryo-EM that a template/primer DNA substrate with a pre-existing mismatch cannot enter the exo site of Pol epsilon/PCNA holoenzyme, but a mismatch generated in the Pol site yields three proofreading intermediates of Pol epsilon/PCNA holoenzyme. These intermediates reveal how the mismatch is dislodged from the Pol site, how the DNA unwinds 6 base pairs and how the unpaired primer 3′-end is inserted into the exo site for cleavage. These results unexpectedly demonstrate that PCNA imposes strong steric constraints that extend unwinding and direct the trajectory of mismatched DNA, and that this trajectory is dramatically different than for Pol epsilon in the absence of PCNA. These findings suggest a physiologically relevant proofreading mechanism for the human Pol epsilon/PCNA holoenzyme.

## Introduction

**Fig. 1.**
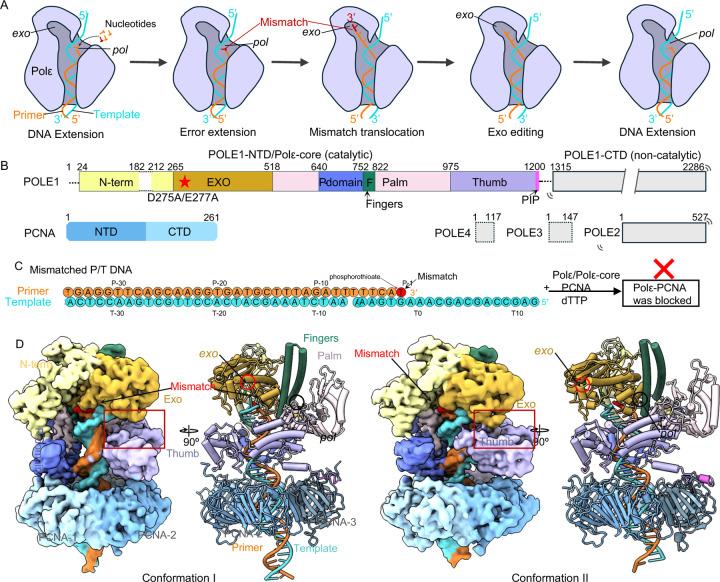
Cryo-EM structures of Polε–PCNA bound to T/P with a pre-existing mismatch. (A) sketch of the Polε holoenzyme proofreading process based on published studies in the absence of sliding clamp. The T/P unwinds by 3 bp and the unwound regions are kept separated during mismatch editing. The *pol* and *exo* sites are labeled. (B) Domain architectures of PolE1–4 and PCNA. Dashed lines indicate disordered regions. The structurally resolved regions (POLE1-NTD, i.e., Polε-core, and PCNA are colored by domains. POLE1-CTD and POLE2–4 are mobile relative to POLE1-NTD and invisible in EM maps. (C) The nucleotide sequence of the primer and template DNA. The mismatched primer 3’-end is highlighted in red. The T/P with a pre-existing mismatch cannot reach the *exo* site of Polε holoenzyme and binds Polε in a blocked state. (D) EM maps and atomic models of the Polε-PCNA–T/P complex in two blocked conformations. The maps and models are labeled and colored by domains as in (B). The distinct interactions of the Exo and thumb domains in the two conformations are highlighted by the red box.

DNA replication is a fundamental process required of all cell types (1–3). Replicative polymerases, such as the leading strand Polε and lagging strand Polδ synthesize DNA with extraordinarily high fidelity (4–7). The high accuracy is achieved by their 3′−5′ exonuclease activity (8–10), which correct errors when they are made by the DNA polymerase (pol) (Fig. 1A). Human Polε belongs to the B-family of DNA Pols and contains 4 subunits, the large catalytic subunit POLE1 (Pol2 in yeast) and three regulatory subunits POLE2–4 (Dbp2-4 in yeast) (11, 12). *POLE1* has undergone a gene fusion between two distinct but related B family DNA polymerases (13, 14). The fusion protein contains a catalytic N-terminal domain (NTD) and a noncatalytic C-terminal domain (CTD) (Fig. 1B); the NTD and CTD are linked by a long loop that interacts with the two small histone-fold POLE3 and POLE4 subunits (15). The CTD is unique to POLE1 (16, 17), and together with POLE2, forms a complex with the replicative helicase Cdc45-Mcm2-7-GINS (CMG) to produce the leading strand replisome (18–21). The POLE1 NTD harbors both DNA polymerase and 3′−5′ proofreading exonuclease (Exo) activities(22, 23), and is therefore referred to as Polε-core in this study.

The Polε-core contains the conserved thumb, fingers, and exo domains plus a unique P-domain (23) (Fig. 1B). Polε-core interacts with the ring-shaped PCNA sliding clamp to assemble the holoenzyme. We and others recently solved the structures of the Polε-core–PCNA–DNA ternary complex in the DNA polymerizing state, revealing that Polε-core contacts all three subunits of the PCNA trimer via its C-terminal PIP motif, thumb domain, and P-domain, respectively, and that the P-domain contributes to Polε processivity by binding to both PCNA and dsDNA (24–26). These structures combined with previous activity assays underscore the importance of the PCNA sliding clamp in Polε activity (27, 28), and are consistent with the functional requirement of a sliding clamp of all other replicative polymerases (29, 30). While the canonical RFC PCNA clamp loader can load PCNA onto either strand (31–33), we recently discovered that PCNA loading by the leading strand specific CTF18-RFC loader is facilitated by the Polε P-domain by stabilizing CTF18-RFC for PCNA opening and DNA loading (34).

Proofreading occurs after the DNA Pol incorporates a mismatched nucleotide in the *pol* site, followed by unwinding a short segment of template/primer (T/P) to enable the 3’ end of the primer to relocate at a distant *exo* site for mismatch removal. Single-molecule analysis showed that Pol alone, without its sliding clamp, can rapidly dissociate from the mismatched DNA at the pol site and rebind DNA at its exo site during in vitro replication (35, 36), but a dissociation-and-reassociation mechanism is not expected for a processive Pol–clamp complex that binds DNA without dissociation (i.e. processive action). In other words, to capture authentic proofreading intermediates as expected to occur in vivo, an in vitro system must minimally ensure that the Pol is attached to its sliding clamp, from misincorporation in the Pol site to excision in the exo site. We find here that Polε misincorporation and proofreading can occur within the Polε–PCNA complex. Interestingly the processive action of Polε confined by PCNA-DNA imposes an unexpected, but important constraint on how the enzyme and DNA move during proofreading.

To ensure that the mismatch in fact occurs at the *pol* site before the mismatch enters the *exo* site, the mismatch should be generated in situ by allowing mismatch formation to proceed to proofreading. If instead one were to provide a preformed mismatch DNA to Polε, it will directly bind the *exo* site without first going through the *pol* site, and the result may not reflect the in vivo situation in which the mismatch originates in the Pol site. This is important because the spatial and temporal coupled processes of the T/P unwinding and *pol*-to-*exo* translocation might be expected to occur in the cell as a processive Polε–PCNA complex.

Several DNA Pol structures, without their cognate clamp, have been reported in the proofreading states, including *E. coli* Pol I (37), *E. coli* Pol III (38), bacteriophage RB69 Pol (39–41), archaeal PolB (42), mitochondrial Polγ (43, 44), and human Polε (25). These structures have suggested a largely conserved proofreading mechanism in which a mis-incorporated nucleotide at the primer 3’ end fails to pass the Watson-Crick base-pairing checkpoint in the *pol* site and is blocked from further polymerization (Fig. 1A). This triggers Pol backtracking, primer unwinding 3 base pairs (bp) from the template, and primer translocation to the *exo* site, where the mismatch is removed (45, 46). The excised primer then translocates back to the *pol* site to resume DNA synthesis. The *E. coli* replicase, DNA Pol IIIα (with its clamp), was also found to unwind 3 bp of the T/P (47), and archaeal PolD-PCNA unwinds 5 bp (48). But all these studies used either Pol in the absence of a sliding clamp or used pre-synthesized mismatch DNA in which a clamp may not encircle DNA during the entire process of mismatch formation and excision. In a recent study of human Polε proofreading, the in vitro system lacked a stably bound PCNA and used a preexisting DNA mismatch (25). Considering Pol proofreading studies thus far lacked a stably bound PCNA, the proofreading mechanism of a processive DNA Pol-clamp complex is not yet known and is a goal of the present study.

In the current study, we use a sufficiently long DNA to stabilize the PCNA clamp and generate the mismatch in situ. In the presence of the PCNA clamp, we find unique results not observed before for any Pol, including Polε lacking PCNA. We first demonstrate by cryo-EM that a T/P with a preexisting mismatch is blocked from entering either the *pol* site or the *exo* site of the human Polε–PCNA holoenzyme. We capture three authentic proofreading intermediates only when a mismatch is first produced in situ at the *pol* site of human Polε–PCNA holoenzyme. These intermediates represent the mismatch-locking state, Pol-backtracking state, and mismatch-editing state. In contrast to the 3-bp melting by the conserved B-family DNA Pol alone or Polε in the absence of PCNA (10, 25), we observe 6 bp are melted by the processive Polε–PCNA. In all three intermediate states, Polε-core is stably associated with PCNA similar to the DNA synthesizing mode. This study is the first in which proofreading intermediates are captured in the context of a functional holoenzyme, and the observed movement of mismatched primer 3’-end is actually originated from the *pol* site. We therefore suggest that the proofreading mechanism revealed by these intermediates may be the physiologically relevant process inside the cell.

## Results

### 1. Polε holoenzyme with a pre-existing mismatch could not enter a proofreading state

A T/P DNA substrate with a 20–23 bp dsDNA region is sufficient to span the human Polε and PCNA in the polymerizing state, but not long enough to maintain stable PCNA binding in the proofreading state (24, 25), because Polε backtracks and unwinds several bp during proofreading (25). We therefore designed T/P substrates with a 35-bp dsDNA region to investigate the proofreading mechanism by the human Polε–PCNA holoenzyme (Fig. 1C). We first asked if a T/P containing a pre-existing mismatch (G•T) can form the expected proofreading intermediates by the Polε–PCNA holoenzyme. We mixed the purified human Polε, PCNA, and the mismatched T/P DNA at a molar ratio of 1:3:1.1 in the presence of 0.5 mM dTTP at room temperature for 10 minutes and incubated the mixture on ice for 2 hours prior to preparing cryo-EM grids (*SI Appendix*, Fig. S1A). Cryo-EM imaging and associated 2D and 3D classifications and 3D reconstruction resulted in two 3D EM maps of the Polε–PCNA–DNA ternary complex at an average resolution of 3.88 Å and 3.81 Å, respectively (Fig. 1D, *SI Appendix*, Fig. S1 and S2).

Atomic modeling of the two maps shows that Polε-core stably binds to the PCNA and P/T, but the POLE1-CTD and the regulator subunits POLE2–4 are invisible (*SI Appendix*, Fig. S1B–H), similar to Polε holoenzyme in the polymerizing state (24, 25). This is consistent with the knowledge that the two Pol lobes of Polε are flexibly linked(19–21). Notably, the template strand extends to the exo site in both structures (Fig. 1D, *SI Appendix*, Fig. S2), indicating that the T/P with a pre-existing mismatch cannot reach the proofreading state in the Polε holoenzyme when the DNA movement is highly constrained by PCNA. Instead, the T/P junction binds just below the Exo and N-term domains in the highly positively charged main substrate chamber (*SI Appendix*, Fig. S2), thus placing the holoenzyme in a blocked state.

The Exo domain is distant from the thumb in one conformation but interacts with the thumb via a thumb-domain-contacting loop (TCL) in the other conformation (*SI Appendix*, Fig. S1A–B). Therefore, the template strand passes through a gap between the TCL and the thumb domain in the first conformation but is blocked by the TCL in the second conformation. While the upper end of the T/P DNA substrate is blocked by Polε, the bottom dsDNA end of the T/P protrudes from PCNA by 6 bp (Fig. 1D). As described below, in the authentic proofreading state, no stable dsDNA density is observed below the PCNA, as the T/P moves upwards and is unwound for proofreading.

### 2. Polε enters an authentic proofreading state upon encountering an in-situ mismatch

**Fig. 2.**
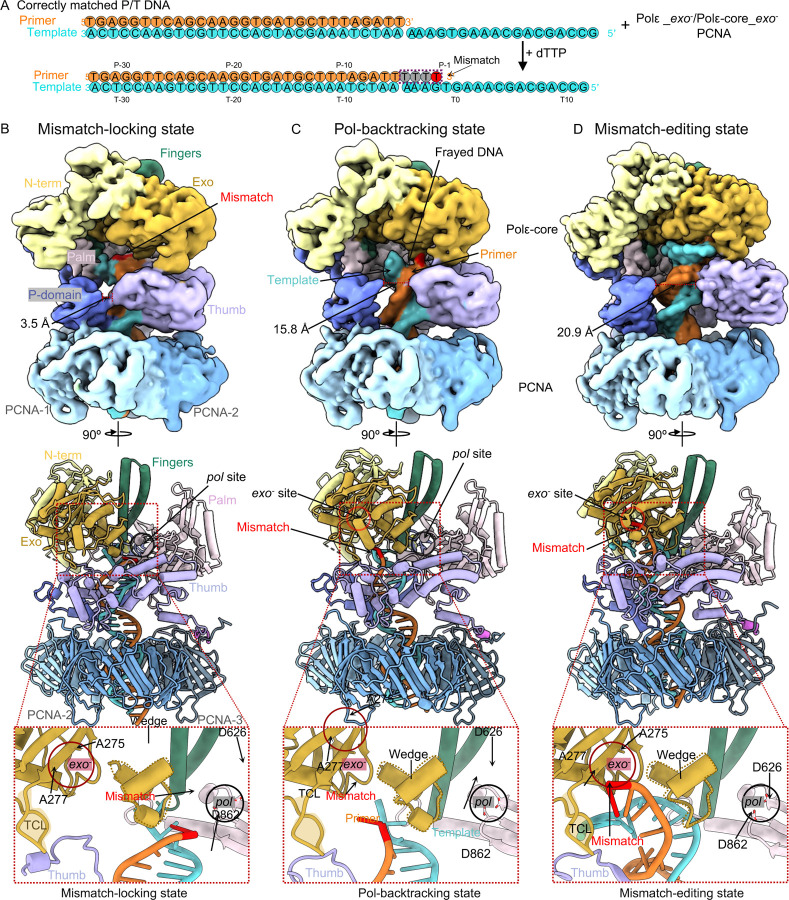
Cryo-EM structures of Polε–PCNA bound to an in-situ generated mismatch. (A) Scheme for capturing Polε holoenzyme in the proofreading states. Nucleotide sequence of the P/T is shown in color. In the presence of dTTP, Polε extends 4 T; 3 are matched with template (gray) and the last one is mismatched (red). (B-D), Cryo-EM maps and atomic models of the ternary complex in the mismatch-locking (B), Pol-backtracking (C), and mismatch-editing (D) states. Cryo-EM maps are shown in the upper panel, atomic models in the middle, and close-up views of the mismatched primer 3’-end in the three proofreading intermediates in the lower panel.

We next designed a T/P substrate lacking a mismatch. The DNA substrate contains a 29-bp duplex region to ensure stable PCNA binding during Polε proofreading and an 18-nt tail in the template strand (AAAGT-GAAAC-GACGA-CCG) (Fig. 2A). In the presence of dTTP, the primer will be extended by three nucleotides (3 T’s) by Pol, followed by a G•T mismatch due to the lack of dCTP. Therefore, this experimental design results in a mismatch that is generated in situ within the *pol* site (44, 49), ensuring that the subsequent movement of the mismatch actually originates from the *pol* site, in a manner that mimics in vivo proofreading.

We initially pre-incubated a Polε Exo^−^ variant (D275A/E277A(50); Fig. 2A), PCNA, and the above-described P/T DNA substrate at a molar ratio of 1:3:1.1, then added 0.5 mM dTTP to the reaction and incubated the mixture at room temperature for 3 minutes prior to making cryo-EM grids. But the assembled complex particles had preferred orientation issues that prevented us from obtaining a good 3D map. Considering that only the catalytic Polε-core binds to PCNA (24, 25), we substituted Polε-core Exo^−^ in place of full length Polε (we note that the proofreading activity of Polε-core is comparable to that of full-length Polε (51).

The in vitro assembled complexes were heterogeneous, as expected for different stages of the proofreading process. By 3D classification and 3D reconstruction, we obtained three 3D EM maps of the Polε-core–PCNA–DNA ternary complex at an average resolution of 3.60 Å, 3.53 Å, and 3.11 Å resolution, respectively (Fig. 2B–D, *SI Appendix*, Fig. S3). This process provided three atomic models (Fig. 2B–D, *SI Appendix*, Fig. S4). In all three structures, the Polε-core fingers domain is in the open conformation such that the Pol activity is arrested, and the primer 3′-end is being transferred from the *pol* to *exo* site, clearly indicating that these states represent proofreading intermediates of the holoenzyme. Importantly, Polε-core stably binds to PCNA via its P-domain, thumb domain, and the C-terminal PIP motif (Fig. 2B–D), in a manner that largely resembles the PCNA interaction when the holoenzyme is in the DNA polymerizing states(24, 25). Therefore, these new structures clearly show that PCNA remains stably associated with Polε and will strongly constrain T/P movement due to PCNA encirclement of DNA. This also limits the movement of the 3’ terminal mismatch relative to the enzyme. We therefore observe very different proofreading behavior of the holoenzyme compared to a previous study performed in the absence of PCNA (25), as detailed below.

### 3. Three proofreading intermediates of the human Polε

Here we describe the DNA binding mode in each of the three proofreading states, with a focus on the location of the in situ synthesized primer 3′-end mismatch.

The first intermediate is a “mismatch-locking state”. The overall Polε-PCNA structure in this state is nearly identical to the polymerizing state of Polε-PCNA (24, 25), except the fingers switch from a closed to an open conformation (*SI Appendix*, Fig. S5A–B). Consistently, the pol site in the first intermediate lacks an incoming nucleotide (Fig. 2B), and the 3′ mismatched primer end is 1 bp below the post-insertion site of Polε (*SI Appendix*, Fig. S5C). Previous studies reveal that the incoming nucleotide cannot be incorporated into the 3′ mismatched primer end due to a failure of Watson-Crick base checking in the post insertion site of DNA Pol (52–54). Thus, when the post-insertion site adopts a 3′ mismatch base, the polymerizing state could mimic the mismatch sensing state. Compared to these states, the holoenzyme has moved forward by 1 bp while maintaining tight binding to the T/P in the first state (Fig. 2B–C, *SI Appendix*, Fig. S5C). Consequently, the mismatch is locked 1 nucleotide below the pol site and cannot be extended by Pol activity. For this reason, we have referred to the first state as the mismatch-locking state in which the *pol*-to-*exo* transition has initiated. Interestingly, when Polγ (a family D Pol) synthesized a mismatch in situ, it also moved forward by 1 bp after mismatch sensing (44).

The second intermediate is in a Pol-backtracking state. In this state, the EM density of the T/P DNA substrate is weaker and frayed such that the unwinding has started (Fig. 2C). Because the unwound primer 3′ mismatch is at the entry of the channel leading towards the *exo* site (Fig. 2C), this state represents the Polε-backtracking state in which initial melting has allowed the primer 3′-end to move away from the pol and toward the exo site. Further, the thumb, along with the dsDNA region, has moved away from the P-domain to facilitate the T/P fraying (Fig. 2B–C).

The third intermediate is in a mismatch-editing state. The T/P DNA substrate has stronger EM density in this state than in the first and second states, indicating it is a relatively stable state of the holoenzyme (Fig. 2D). The mismatched primer 3′-end has fully translocated into the *exo* site (Fig. 2D). In a wild-type Polε, the mismatch would have been cleaved in this state (8), but because we used the mutant Polε exo^−^ in this study there is no cleavage.

### 4. Mismatch translocation from the mismatch-locking to Pol-backtracking state

**Fig. 3.**
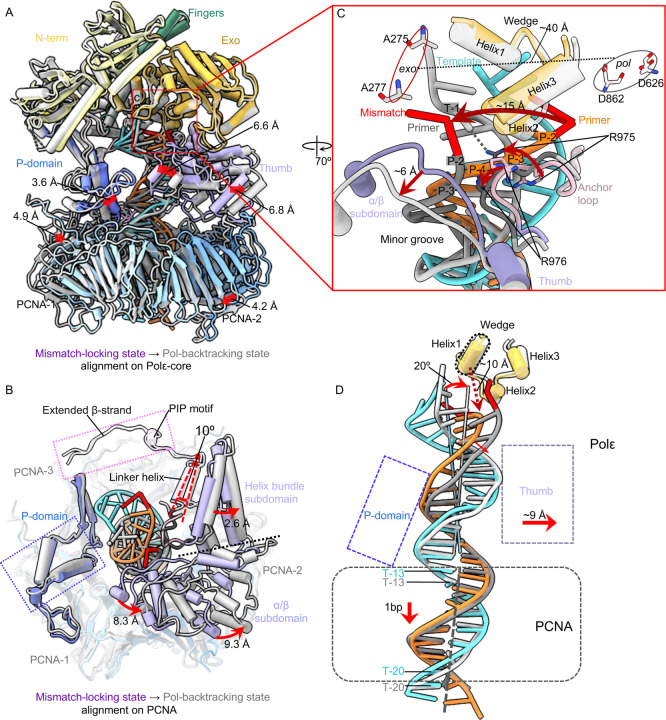
Conformational changes of the T/P from the mismatch-locking to Pol-backtracking state. (A) Superimposition on Polε-core of the mismatch-locking (color) and Pol-backtracking states (gray). Domain movements are indicated by red arrows. (B) A top view of the superimposition at the Polε-PCNA interface where most movements occur. The bulk of Polε-core is omitted for clarity. (C) Close-up view of changes in the mismatched region of T/P between the two states. The movements are indicated by red arrows and labeled. (D) Superimposition of T/P based on alignment on PCNA in both states. The DNA tilts 20° and moves with the thumb outwards by 9 Å. And the DNA moves down by 1 bp.

Superimposition of the Polε-core backtracking and mismatch-locking states reveals that the P-domain and the α/β subdomain of the thumb moves away from the dsDNA region by 3.6 Å and 6.8 Å, respectively (Fig. 3A). The larger space between the P-domain and the thumb allows a longitudinal movement of the T/P by 1 bp in the backtracking state. The PCNA undergoes a rigid-body movement, but the interaction between the P-domain and PCNA-1, and between the PIP motif with the extended β-strand and PCNA-3, remain unchanged (Fig. 3B). These interactions resemble the corresponding interactions between Polε-core and PCNA in the polymerizing state(24, 25) (*SI Appendix*, Fig. S5D). However, the α/β subdomain of the thumb, which only interacts with PCNA very weakly, moves outwards by ~ 8–9 Å above PCNA-2. And this leads to a 2–3 Å outward movement of the helix-bundle subdomain of the thumb, which in turn, results in a ~10° outward rotation of the linker helix connecting the PIP motif and the helix-bundle subdomain (Fig. 3B). Overall, the three-point interaction between Polε-core and PCNA is largely sustained transitioning from the mismatch-locking to Pol-backtracking state.

The *pol* and *exo* sites are ~40 Å apart (Fig. 3C). The mismatched primer 3′-end moves about 15 Å from the locking to backtracking state, and the primer ends in these two states are separated by a three-helix wedge in the Exo domain. It appears that the inner α/β subdomain of the thumb has pushed the primer at the P-2/P-3 region to a distinctive position in the Pol-backtracking state. Further, the loop linking the palm and thumb domains contains multiple positively charged residues that may anchor the T/P transition during proofreading (25, 51). For example, Arg-975 and Arg-976 seem to reposition the primer at the P-3/P-4 region, and both residues are inserted in the T/P minor groove (Fig. 3C). Importantly, Arg-975 appears to directly contribute to the T/P fraying by forming a H-bond with the template T-1 base, which enables the mismatched primer 3’-end to flip toward the *exo* site in the Pol-backtracking state. The dsDNA region inside PCNA slides down by 1 bp from the mismatch-locking to the backtracking state (Fig. 3D), and the dsDNA region inside the Polε-core tilts 20° toward the thumb domain, which moves away by ~9 Å to accommodate the new DNA location. The wedge helix1 is 10 Å above the frayed T/P junction and is likely not responsible for the T/P separation, in contrast to the suggested Polε proofreading model (25). Overall, the thumb and the anchor loop drive the T/P transition from the locking to backtracking state.

### 5. Polε holoenzyme in the mismatch-editing state

**Fig. 4.**
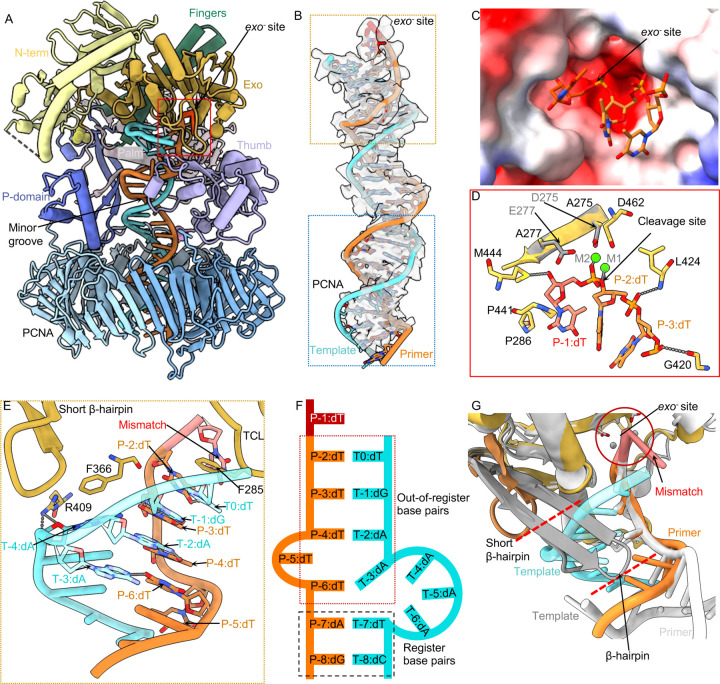
Polε interactions with T/P DNA in the mismatch-editing state. (A) The mismatch-editing state structure colored by domains. (B) T/P DNA in sticks superimposed with the EM density in transparent surface view. The top orange box shows the clear DNA density around the *exo*^-^ site of Polε, the bottom blue box shows that the weak DNA density inside the PCNA clamp. (C) Electrostatic surface of the *exo*^-^ site with the primer 3′-end. (D) The Polε *exo*^-^ site structure. The catalytic D275 and E277 coordinating two Mg^2+^ in wild-type Polε are shown; they are mutated to A275 and A277 in Polε-exo^−^. (E) Close-up view of T/P around the *exo*^-^ site. The T/P melts 6 bp then forms 4 out-of-register base pairs. The β-hairpin does not contact the template. Residues stabilizing the T/P are in sticks and labeled. (F) Sketch of T/P in editing state. (G) Comparison of the short Polɛ β-hairpin of (dark orange) and the longer RB69 gp43 β-hairpin (gray). The longer phage β-hairpin separates the template from primer.

The primer 3′-end reaches the *exo* site in the mismatch-editing state (Fig. 4A). The upper region of the T/P has well-defined EM density that resolves individual bases (Fig. 4B). There are three separated primer bases in the *exo* channel, similar to previous studies on B-family DNA Pols (25, 40, 55, 56) (Fig. 4C–D). The phosphate of the 3′-end P-1:dT faces a highly negatively charged region of the exo site, which would otherwise be mediated by two cations in the wild type exo site. But the 3′ dT ribose hydroxyl H-bonds with Met-444, and the P-1:dT base form a hydrophobic interaction with Pro-286 and Pro-441 (Fig. 4C–D). These interactions may be sufficient to stabilize the primer 3′-end in the mutated *exo* site (*exo*^-^). Superimposing the wild type exo site (PDB ID 9F6L(25)) with the current exo^−^ site reveals that the two catalytic Mg^2+^ ions align precisely to coordinate the phosphate of the mismatched P-1:dT (Fig. 4D). The following P-2:dT/P-3:dT region is stabilized by two H-bonds, with their respective phosphate H-bonds with Leu-424 and Gly-420, respectively (Fig. 4D).

A total of six bp are melted from the primer 3′-end in the editing state of the holoenzyme, the longest unwound stretch observed to date in any proofreading study (Fig. 4E–F). Unexpectedly, the template strand is scrunched by 2–3 nucleotides, such that the frame-shifted and melted template and primer form two mismatched base pairs (P-2:dT with T0:dT, P-3:dT with T-1:dG) and two serendipitously correct base pairs (P-4:dT with T-2:dA and P-6:dT with T-3:dA). But P-5:dT does not base-pair with the template (Fig. 4E–F). Therefore, the P-2 and P-3 bases in the exo channel are protected by the out-of-register base pairing, allowing only the mismatched primer 3’-end to be cleaved. The Polε exo domain adopts the DnaQ-like exonuclease fold conserved among A, B, and C-family DNA Pols(57). While all B-family DNA Pols possess a β-hairpin loop in the exo domain to facilitate T/P translocation between the *pol* and *exo* sites(55, 58, 59), the β-hairpin loop is much shorter in Polε (51) (Fig. 4G). The structure of the mismatch-editing state reveals that the β-hairpin is too short to interact with the template and to keep it separated from the primer (Fig. 4E,G). Instead, the template is only stabilized at T-4:dA by the Exo domain Arg-409 forming two H-bonds and the aromatic Phe-366 via a π-π interaction (Fig. 4E). We therefore suggest that the uniquely short β-hairpin loop underlies the out-of-register base-(mis)pairing in the T/P. Importantly, it is Phe-285 of the exo domain TCL that stabilizes the T0:dT via a π-π interaction to keep the template strand separated from the primer 3′-end (Fig. 4E). This perhaps explains why the neighboring residue Pro-286 is so important to the proofreading activity and that the P286K/R mutations are often found in cancers (50, 60, 61).

Beyond the exo domain, the Polε-core P-domain, fingers, palm, and thumb domains primarily interact with the dsDNA region (*SI Appendix*, Fig. S6). Specifically, the P-domain Arg-672, Glu-674, Lys-733, and His-735, the fingers domain Lys-822, the palm domain Lys-950 and Lys-953, and the anchor loop Lys-974 and Arg-976 all interact with the duplex. In the thumb α/β subdomain, Ser-1038 engages the phosphate, and Ser-1040 H-bonds with thymidine base of P-4:dT (*SI Appendix*, Fig. S6A). The DNA duplex is slightly tilted towards PCNA-1 in the PCNA chamber and is surrounded by positively charged residues such as PCNA-1 Lys-14, Lys-20, Lys-77, Arg-149, and Lys-217, PCNA-2 Lys-80 and Arg-149, and PCNA-3 Lys-80 and His-153 (*SI Appendix*, Fig. S6B). But these residues are 4–6 Å away from the duplex phosphate backbones to allow DNA mobility, likely accounting for the weaker DNA density inside the PCNA ring (Fig. 4B).

### 6. Changes in Polε during transition from the Pol-backtracking to mismatch-editing state

**Fig. 5.**
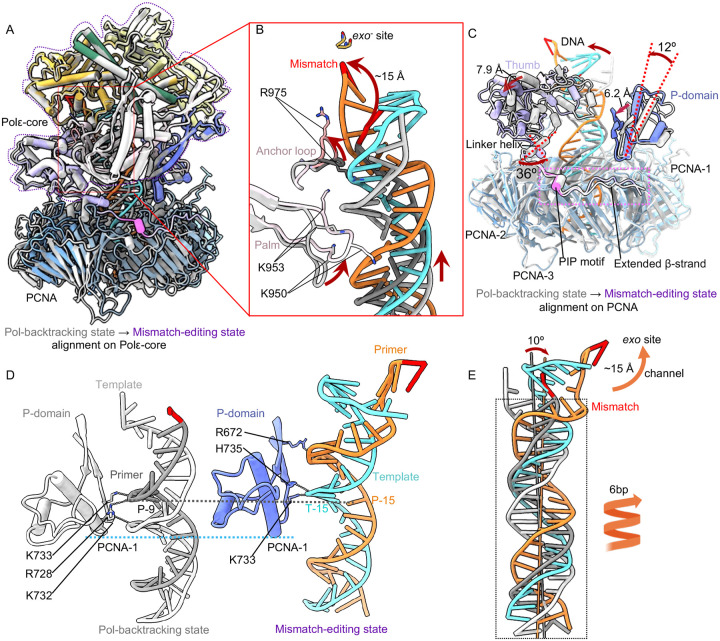

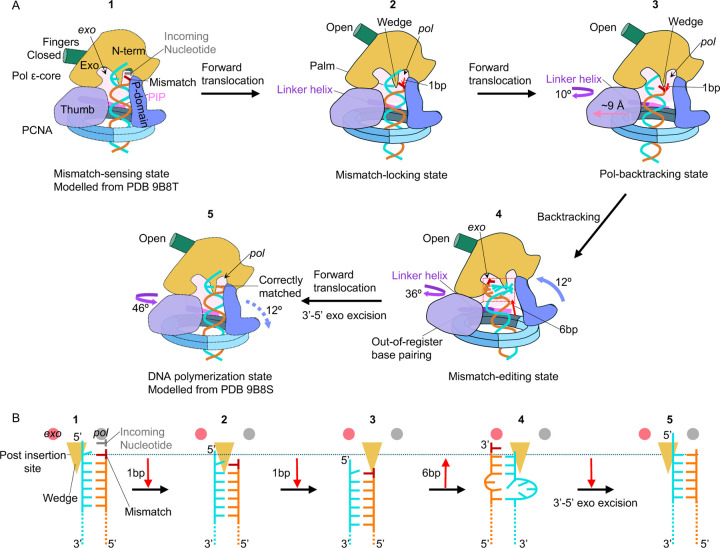
Conformational changes of Polε–PCNA induce the mismatched DNA translation during backtracking. (A) Comparison of the Pol-backtracking (gray) and mismatch-editing (color) states aligned on Polε-core. (B) Close-up view of the T/P and surrounding region. Red arrows indicate movements between the two states. Key residues are in sticks and labeled. (C) Structural changes near the Polε-PCNA interface. The top region of Polε-core is omitted for clarity. (D) Side-by-side comparison of the P-domain and T/P interaction in the two states. DNA-interacting residues are in sticks and labeled. (E) Comparison of the T/P in the two states reveal an overall translocation. The DNA tilts 10° toward the *exo* activity site. The duplex region spirals up by 6 bp, and 6 bp are unwound from the top primer 3′-end, enabling the primer to move 15 Å through exo channel into the *exo* site.

The T/P undergoes dramatic transformation, as only one base at the mismatched primer 3′-end is frayed in the Pol-backtracking state, but six base pairs are melted from the primer 3′-end and rearranged in the mismatch-editing state (Figs. 3–4). Comparison of these two states reveals that the top region of Polε-core is well aligned but its PCNA contact region undergoes substantial conformational changes (Fig. 5A). Specifically, the palm domain loop moves (from orange and blue) to engages DNA with its Lys-950 and Arg-953, after transition to the mismatch editing state (Fig. 5B). And the anchor loop (primarily Arg-975) that originally inserted in the DNA minor groove (between white and gray), now flips upward and dissociates from DNA (between orange and blue) in the mismatch editing state (Fig. 5B). Because the local DNA movement directions coincide with the movement directions of both the palm and anchor loops as described above, we suggest that the T/P transformation is driven by these two Polε-core loops.

The interfaces between the Polε P-domain and PCNA-1 and between the Polε PIP motif plus the extended β-strand with PCNA-3 are maintained during the transition. But the thumb domain shifts laterally by 7.9 Å above PCNA-2. This large shift is driven by a ~36° rotation of the linker helix connecting the thumb and the PIP motif. The P-domain tilts 12° toward DNA by pivoting on the bottom region of Polε that contacts PCNA, leading to a 6.2 Å shift of the P-domain β-strands such that they interact with the DNA minor groove (Fig. 5C). Thus, the bottom region of the Polε P-domain binds DNA in the Pol-backtracking state but it is the upper region of the P domain β-strands that bind DNA in the mismatch-editing state (Fig. 5D). Importantly, this moves the DNA up along with the P-domain during the transition correlating with the observed extensive unwinding. Correspondingly, Lys-733 interacts with the P-9 base in the Pol-backtracking state and with the T-15 base in the mismatch-editing state (Fig. 5D), consistent with the DNA spiraling up by 6 bp during the transition as well as 6-bp melting and rearrangement in the editing state (Fig. 5E). During the transition, DNA tilts by 10° and the Polε–PCNA holoenzyme backtracks, enabling the mismatched primer 3′-end to move by 15 Å to reach the *exo* site (Fig. 5E). These actions result in twice the unwinding observed in Pol editing modes in the absence of connection to processivity clamps.

## Discussion

Recent studies on the polymerizing state of Polε holoenzyme have shown that Polε-core engages with PCNA through a three-point interface, interacting with all three protomers of the PCNA clamp(24–26). However, it has been unclear if PCNA remains fully engaged during Polε proofreading, and if so, how the sliding clamp constrains the T/P movement while allowing the primer 3′-end to travel the long-distance from the *pol* to the *exo* site. PCNA is more than a processivity factor and is an integral part of a replicative Pol holoenzyme. Therefore, PCNA is expected to play an important role in the proofreading mechanism. But in the recent cryo-EM analysis of Polε proofreading, PCNA was largely dissociated from Polε in the presence of a pre-existing mismatched T/P with a 23-bp dsDNA region (25). The dissociation could be part of the proofreading mechanism to confer more freedom to Polε relative to the T/P as they negotiate the *pol*-to-*exo* transition, but Polε dissociation from PCNA might have been caused by the short duplex region of the T/P substrate used in that study. In the current study we used a longer duplex region and observed a 6-bp unwinding during Polε backtracking, and Polε remains attached to PCNA. Indeed, the shorter duplex region used in the previous study would not have been able to observe this consistent Polε–PCNA contact. Considering that the major interactions between Polε-core and PCNA observed in the polymerizing state are in fact retained throughout the proofreading process when using the longer duplex in this study, it seems likely that the proofreading process occurs as described in this report, although it is possible that there exist multiple proofreading pathways.

### *Bona fide* proofreading requires both PCNA and *in-situ*-generated mismatch

Remarkably, we found that the tightly coupled PCNA imposes such strong constraints on Polε that a T/P with a pre-existing mismatch could not insert from outside of the enzyme into the exo site. This observation calls into question the previous structural studies on the proofreading mechanism of other DNA polymerases that are known to function with a DNA sliding clamp (39–44).

We succeeded in capturing the proofreading states in the context of the holoenzyme only when the mismatch was synthesized in situ in the *pol* site of Polε. To our knowledge, this is the first time that DNA polymerase proofreading intermediates have been visualized in a near native environment in which the polymerase is complexed with the sliding clamp and the mismatch is first produced inside the *pol* site and then travels to the *exo* site. Under these more physiologically relevant conditions, we found that the human Polε proofreading mechanism is strikingly different from previous observations using preformed mismatched DNA that does not require Pol–clamp complex.

### Proposed proofreading process by the human Polε–PCNA holoenzyme

**Fig. 6.**
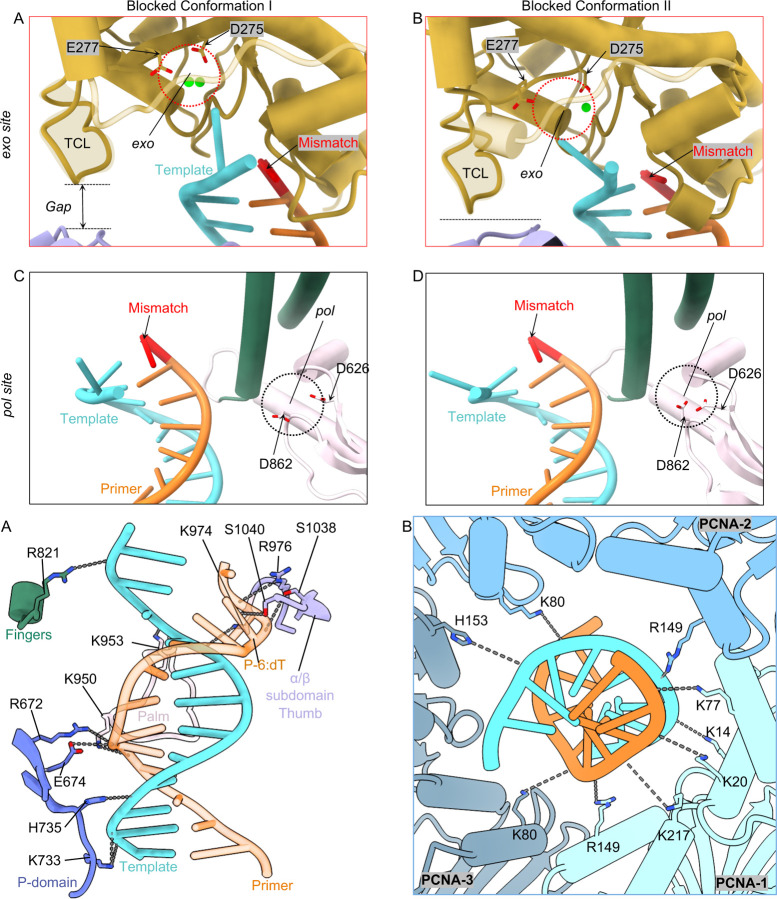
Model of the Polε–PCNA holoenzyme proofreading process. (A) Schematic representation of all five steps that are built based on experimental structures of the human Polε–PCNA holoenzyme. In step 1, Polε incorporates a mismatched base in the *pol* site and senses the mismatch via a Watson-Crick base-pairing checkpoint. This is modeled by Polε structure in the Pol state (PDB ID 9B8T). In step 2, Fingers domain flips up to an open position to arrest the pol activity, and the holoenzyme moves away from the mismatched 3′-end by 1 bp to prevent additional base incorporation. In step 3, the Polε linker helix rotates 10° and the thumb domain moves outwards by 9 Å. The movements put pressure on the 1-bp backtracked T/P. In step 4, the P-domain tilts 12° against the PCNA, and the linker helix rotates 36°, causing the holoenzyme to backtrack by 6 bp and unwind 6 bp from the primer 3′-end. The 6 unwound bp rebind and form 4 out-of-register bp and to insert the mismatched primer 3′-end into the *exo* site. In step 5, the primer 3′ mismatch is excised by the exo activity. Next (not shown), in an expected reverse process not yet well characterized, the holoenzyme returns the T/P to the *pol* site and resumes DNA synthesis. (B) Schematic describing the mismatch DNA translocation during the Polε–PCNA holoenzyme proofreading process. The horizontal dashed line indicates the post insertion site of Polε. The movement of DNA between each step is indicated by red arrows.

Morphing the three proofreading intermediates observed here provides a plausible view of the *pol*-to-*exo* translocation process of a mismatched T/P DNA that is made in the Polε active site and is then transferred to the exo site for removal of the mismatch (**Movie S1**). We therefore propose the following proofreading mechanism for the human Polε (Fig. 6). It is known that proofreading is initiated (step 1) when a mismatched base is synthesized at the *pol* site and fails the Watson-Crick fidelity check, and there have been studies on how this fidelity check might work (52–54). But for the earliest state captured in this report Polε has shifted forward by 1 bp on the template and thus has passed the checkpoint step, and is therefore at step 2, the mismatch-locking state (Fig. 6). Due to the T/P having backtracked from the *pol* site by 1 bp, the fingers domain is now switched to the open conformation (Pol inactive). This 1-bp-mismatch displacement is important for preventing potential Pol extension on the mismatch. In step 3, Polε rotates slightly above the PCNA, accompanied by a 10° outward rotation of the linker helix between the PIP motif and the thumb domain, and a 20° tilt of the DNA to shift the thumb domain outwards by 9 Å, making room for DNA translocation (Fig. 3B). These changes result in the anchor loop that appears involved in separating the primer 3’-end from template (51). In step 4, the Polε P-domain tilts by 12° and the linker helix rotates by 36° to drive T/P unwinding by 6 bp and move the mismatched primer 3′-end by 15 Å towards the *exo* site (Fig. 5C–E). The 6-bp T/P melting is the longest unwinding for proofreading observed thus far, as previous studies observed melting of only 3 bp during proofreading in Polε (25) and other B-family DNA polymerases (39–44). In the last step, step 5 which is not observed in this study, the mismatched base is expected to be cleaved by the 3′−5′ exo activity before the primer returns to the *pol* site to resume DNA synthesis (Fig. 6).

### Implication of the P-domain involvement in Polε proofreading

The eukaryotic Polε has undergone two remarkable inventions during evolution: formation of the POLE1 catalytic domain fused to another B family noncatalytic POLE1 CTD that tethers POLE to the replicative helicase, and addition of the P-domain in the catalytic NTD. Recent studies have shown that the P-domain enhances Polε processivity by directly binding to DNA and PCNA (23–26), and the P-domain also stabilizes the intrinsically mobile Ctf18 AAA+ domain to help PCNA loading onto the leading strand DNA by the Ctf18-RFC clamp loader (34, 62–64). This study now reveals a third function of the P-domain in T/P melting and repositioning during mismatch proofreading (Fig. 5C–D). Because the P-domain is unique to Polε, this raises the question whether the proofreading mechanism we have described above is unique to Polε. Perhaps other replicative DNA polymerases may use a different proofreading mechanism, possibly by dissociating from their processivity clamp. Structural analysis of other polymerases in the presence of a sliding clamp and use of a mismatch generated in situ will be needed to clarify this question.

### Implications of out-of-register base-(mis)pairing of melted T/P

An important discovery of this study is the out-of-register base pairing or base mispairing of the 6 melted base pairs. In the previously reported Pol proofreading state, 3 base pairs are unwound, and the unwound regions of template and primer are kept separate by the long β-hairpin that protrudes downwards from the exo domain (55, 58). But the corresponding β-hairpin loop is uniquely short in Polε and does not reach the template to keep it from rebinding to the primer, and this out-of-register rebinding leaves only the single mismatched 3′-nucleotide in the *exo* site (Fig. 4E–F). This represents a pre-excision stage of the exo-editing model in which Polε is unable to continue cutting the correct 3′ primer bases. It is currently unclear if such T/P proofreading configuration is adopted in other Pols or whether this process is unique to Polε–PCNA. Regardless, this configuration may be related to the long-recognized but mysterious “replication slippage” phenomenon (65–67). Slippage frequently occurs in DNA regions containing homo-nucleotide runs – stretches of DNA with an identical nucleotide. We speculate that out-of-register rebinding of the melted primer and template regions during proofreading will result in perfect base pairing in a homo-nucleotide run, and such base pairing is harder to break up for realignment (in-register base pairing) when the exo-edited T/P returns to the Pol site. In these cases, failure of T/P realignment might then introduce replication slippage upon resumption of DNA synthesis in the *Pol* site.

This study represents the first revelation of a bona fide proofreading process of the human Polε-PCNA holoenzyme. Mutations in the Polε exo domain compromise replication fidelity and are drivers for tumorigenesis in a wide range of human cancers (68, 69). Our study hence highlights those mutations at the interface between Polε and PCNA may also affect its proofreading activity and could be involved in cancer progression. We also note that the approach taken here, using in situ mismatch formation, can be used to interrogate proofreading mechanisms of many other DNA polymerases.

## Materials and methods

Details on the materials and methods for the expression and purification of human Polε, Polε-core and PCNA are provided in the SI Appendix. As a control for the structural analysis of the in-situ formation of a mismatch, Polε or Polε-core and PCNA were directly mixed with DNA containing a pre-existing mismatch. For in situ formation of a mismatch, the PCNA and Polε were mixed with a flush primed DNA followed by adding dNTPs that result in a mismatch. Only the in-situ method provided the physiologically relevant proofreading intermediates. The detailed methods off assembly, grid formation, data collection, 3D map reconstruction, model building and refinement are provided in the SI Appendix.

## Supplementary Material

1

## Data Availability

Cryo-EM structural data have been deposited in the Electron Microscopy Data Bank (https://www.ebi.ac.uk/pdbe/emdb/) and the Protein Data Bank (https://www.rcsb.org) under the following accession codes: Human Polε–PCNA–T49/P35 (pre-existing mismatch) in the blocked conformation I (EMD-49302 (70) and 9NE9 (71)), human Polε–PCNA–T49/P35 (pre-existing mismatch) in the blocked conformation II (EMD-49303 (72) and 9NEA (73)), human Polε-core–PCNA–T47/P29 (with in situ generated mismatch) in the Mismatch-locking state (EMD-49301(74) and 9NE8 (75)), human Polε-core–PCNA–T47/P29 (with in situ generated mismatch) in the Pol-backtracking state (EMD-49300 (76) and 9NE7 (77)), and human Polε-core–PCNA–T47/P29 (with in situ generated mismatch) in the Mismatch-editing state (EMD-49299 (78) and 9NE6 (79)).

## Significance Statement

Replicative DNA polymerases (Pol) function with a sliding clamp and their proofreading exonuclease provides high fidelity. Thus, study of the proofreading mechanism must both contain the Pol-clamp complex and generate the mismatch in situ to ensure the mismatched primer follows a physiological route from the pol to exo site. Despite numerous previous studies, none satisfy both criteria. This study on human Polε–PCNA meets both criteria. Cryo-EM analysis captured proofreading intermediates that reveal an unexpected proofreading process. The primer is unwound by 6 nucleotides that scrunches to form out-of-register base pairs with the template. These findings provide new insights and calls for reevaluation of proofreading mechanisms performed without a sliding clamp or with an existing mismatch.
