# Supplementary material for: The proofreading mechanism of the human leading strand DNA polymerase ε holoenzyme
Source: bioRxiv. 2025 Apr 12:2025.04.11.648458. Preprint. [Version 1] doi: 10.1101/2025.04.11.648458 (PMC12027367; doi:10.1101/2025.04.11.648458)
Supplement: 1 [file NIHPP2025.04.11.648458V1-supplement-1.pdf]

Supplementary Information Appendix for:

The proofreading mechanism of the human leading strand DNA polymerase ε holoenzyme

Feng Wang [#1], Qing He [#1], Michael E O'Donnell [2*], Huilin Li [1*]

[1] Department of Structural Biology, Van Andel Institute, Grand Rapids, MI, USA
[2] DNA Replication Laboratory and Howard Hughes Medical Institute, The Rockefeller University, New York, NY, USA
[#] These authors contributed equally

* Address correspondence to MEO (odonnel@rockefeller.edu) or HL (huilin.li@vai.org)

This Appendix includes:
Detailed Experimental Procedures
1 Table
1 Supplementary Video legend
6 Supplementary Figures
References for Supplementary Material

Materials and Methods

Molecular cloning and DNA oligomers. The cDNAs of human Polε (POLE1-4) were acquired from DNASU Plasmid Repository, and the cDNA of human PCNA was obtained from Addgene. The cDNA of POLE1(Gene ID: 5426) was tagged with an 6xHis sequence at N-terminus and inserted into the pFL multi-gene expression vector(1). And the cDNAs of POLE2 (Gene ID: 5427, with an N-terminal 3xFLAG tag), POLE3 (Gene ID: 54107), and POLE4 (Gene ID: 56655, with an N-terminal 3xFLAG tag) were cloned into a second pFL multi-gene expression vector(1). The truncated POLE1 (1–1200 aa, Polε-core), containing a tandem N-terminal 3×FLAG-6×His tag, was cloned into the pFastBac vector. The cDNA of human PCNA (Gene ID: 5111) with an N-terminal 6xHis tag was cloned into the pET28a vector. The exo^{-} site mutations (D275A/E277A) of POLE1 or Polε-core were generated by PCR-based mutagenesis. All constructs were sequenced to ensure that mutations were not introduced during PCR and cloning.

The DNA substrates used in the cryo-EM structural studies were chemically synthesized by Eurofins Genomics. The correctly matched T/P DNA substrate (T47/P29) includes a 29-nt primer strand (5′-TGAGGTTCAGCAAGGTGATGCTTTAGATT-3′) and a 47-nt template strand (5′-GCCAGCAG CAAAGTGAAAAATCTAAAGCATCACCTTGCTGAACCTCA-3′). The mismatched T/P DNA (T49/P35) contains a 35-nt primer strand (5′-TGAGGTTCAGCAAGGTGATGCTTTAGATTTTTCA*T-3′) and a 49-nt template strand (5′-GAGCCAGCAGCAAAGTGAAAAATCTAAAGCATCACCTTGCTGAACCTCA-3′).

A phosphorothioate bond (*) was also introduced between the last two nucleotides of the 35-nt primer to prevent cleavage of the mismatched primer 3′-end. For controls, a standard 35-nt primer strand without the phosphorothioate modification was also prepared. But the T49/P35 substrate

with or without the modification led to the same Polε structure. Primer and template oligonucleotides were mixed in equimolar amounts to achieve a final concentration of 100 μM in an annealing buffer (20 mM HEPES, pH 7.5, 50 mM NaCl, and 0.5 mM EDTA). The mixture was heat denatured at 95°C for 10 minutes and then gradually cooled to room temperature. Finally, the annealed P/T DNA were stored at –20°C until use.

Protein expression and purification. Wild-type Polε or Polε exo$^-$, featuring a 6xHis tag at the N-terminus of POLE1 and 3xFLAG tags at the N-termini of POLE2 and POLE4, was expressed using the Bac-to-Bac Baculovirus expression system (Thermo Fisher), following a protocol similar to that used for the EXO mutant of Polε (2). Sf9 cells were co-infected with baculoviruses encoding POLE1 and the POLE2-4 subcomplex and incubated at 27°C with shaking at 115 rpm for 65-72 hours. The harvested insect cells were sonicated in lysis buffer (25 mM HEPES, pH 7.5, 250 mM NaCl, 1 mM MgAc, 5% glycerol, and one tablet of EDTA-free protease inhibitor cocktail). The lysate was centrifuged at 125,440 × g for 1 hour using a Ti-45 rotor, and the supernatant was incubated with 0.8 mL of FLAG antibody-conjugated beads at 4°C for 2-3 hours. After washing the beads twice with 50 mL lysis buffer, the bound proteins were eluted with 8 mL lysis buffer supplemented with 0.2 mg/mL FLAG peptide. The eluted protein was concentrated using an Amicon centrifugal concentrator (100 kDa cutoff) and further purified by size-exclusion chromatography (Superose 6 Increase, GE Healthcare) in buffer containing 25 mM HEPES, pH 7.5, 200 mM NaCl, 1 mM MgAc, and 1 mM DTT. The purified Polε was concentrated to 3.7 mg/mL and stored at –80°C. Polε-core or Polε exo$^-$ was expressed and purified following the same protocol used for full-length Polε. Human PCNA was expressed in *E. coli* BL21 as previously described(3). Briefly, PCNA expression in *E. coli* was induced by 0.3 mM IPTG at 16°C for 18 hours. Cells were harvested and resuspended in lysis buffer (25 mM HEPES, pH 7.5, 200 mM NaCl, 5% Glycerol). Cell lysates were obtained by homogenization using an SPX Corporation homogenizer and subsequently clarified by centrifugation at 34,572 x g for 1 hour at 4°C. His-tagged PCNA was purified using a Ni-NTA column (Cytiva), followed by size-exclusion chromatography on a Superdex 200 column (GE Healthcare) in buffer containing 25 mM HEPES (pH 7.5), 200 mM NaCl, and 1 mM DTT. Purified PCNA was concentrated to 3.0 mg/ml and stored at –80°C.

Cryo-EM sample preparation and data collection. To assemble the *in vitro* ternary complex of human Polε–PCNA–DNA (pre-existing mismatched T/P), we first mixed 1 μM Polε/Polε-core, 3 μM PCNA, 1.1 μM mismatched DNA (T49/P35), and 0.5 mM dTTP at room temperature for 10 minutes and incubated the mixture on ice for 2 hours prior to grid preparation. The simultaneous addition of all three components only produced a ternary complex in the blocked state. We next changed the assembly scheme by first mixing PCNA and DNA, then adding Polε or Polε-core and dNTPs. But this produced the same blocked states. To address the preferred particle orientation issue, we added 0.02% octyl β-D-glucoside (β-OG) into the sample solution immediately before grid vitrification. We did not perform the third assembly scheme of first mixing Polε or Polε-core with mismatched DNA, followed by addition PCNA. Because this mixing scheme was used previously(4) which resulted in Polε/Polε-core interacting with the mismatched DNA prior to PCNA, a scenario we wanted to avoid.

To assemble the ternary complex of human Polε–PCNA with an in-situ-generated mismatched T/P DNA, we mixed 1 μM Polε exo$^-$/Polε-core exo$^-$, 3 μM PCNA, and 1.1 μM correctly-matched DNA (T47/P29) and incubated the mixture on ice for 2 hours, then added 0.5 mM dTTP and incubated the reaction mixture at room temperature for 3 minutes to allow Polε to extend the primer by four bases and generate a terminal G•T mismatched base pair in situ in the *pol* site. For cryo-EM grid preparation, holey carbon grids (Quantifoil Au R1.2/1.3, 300 gold mesh) were glow-discharged in an Ar/O_2 mixture for 30 seconds using a Gatan 950 Solarus plasma cleaner. A 3

μL aliquot of each final reaction mixture containing 0.02% β-OG was applied to the freshly treated grids. Vitrification was performed using a Vitrobot Mark IV system (Thermo Fisher Scientific) with the following settings: blot time of 3 s, blot force of 3, wait time of 5 s, sample chamber temperature of 6 °C, and chamber relative humidity of 100%. Grids were then flash-frozen in liquid ethane cooled by liquid nitrogen.

Cryo-EM data were collected using a 300 kV Titan Krios microscope, operated via SerialEM in multi-hole mode(5). Images were all acquired at 105,000× magnification, with defocus values ranging from –1.2 to –1.6 μm. The data were captured on a Gatan K3 direct electron detector in super-resolution mode, with a pixel size equivalent to 0.414 Å at the specimen level. Each exposure lasted 1.0 second, during which 50 frames were captured, resulting in a total dose of 60 $e^-/Å^2$.

Image Processing and 3D Reconstruction. For the ternary complex of Polε–PCNA-T49/P35 (pre-existing mismatch), a total of 26,913 raw movie micrographs were collected and motion-corrected using MotionCorr 2.0(6) with 2× binning, yielding a pixel size of 0.828 Å/pixel. The motion-corrected micrographs were imported into cryoSPARC (version 4.5.1) for patch-based contrast transfer function (CTF) estimation(7), and 20,493 micrographs with CTF signals extending to 4.5 Å were selected for further processing (**Supplementary Fig. 1d**). Blob-based auto-picking (particle diameters of 100–160 Å) was implemented in cryoSPARC(7) to select initial particle images, which were used to generate a set of 2D classes for the next template-based particle picking. A total of 10,642,983 raw particles were automatically picked and 4× binned. After several rounds of 2D classification, particles with clear structural features were selected. In total, 978,800 selected particles were extracted with a box size of 320 pixels and used to compute five initial 3D models. Three low-quality 3D reconstructions were discarded as junks. One 3D model represented the noncatalytic form of Polε but failed to reach a good resolution upon further refinement. The remaining 3D model had good structural features and was selected for homogeneous and non-uniform 3D refinements, resulting in a 3D map that contained high conformational heterogeneity. Further 3DVA analysis (8) and 3D classifications were performed to generate three subclasses. One subclass was subjected to the Blush regularization refinement(9) to produce an intermediate 3D map at an overall resolution of 4.07 Å. The particles belong to this subclass were then imported into Relion 5 for Bayesian polishing Relion 5 (10), followed by non-uniform refinement in cryoSPARC, leading to a slightly improved final resolution of 3.88 Å. The remaining two subclasses were combined and subjected to Blush regularization refinement, generating an intermediate 3D map at 4.03 Å resolution. Further Bayesian polishing and non-uniform refinement in cryoSPARC improved the resolution to 3.81 Å.

For the ternary complex of Polε–PCNA–T47/P29 with an in situ-generated mismatch, a total of 30,391 raw movie micrographs were collected and motion-corrected using MotionCorr 2.0(6) with 2× binning and an effective pixel size of 0.828 Å/pixel. The motion-corrected micrographs were imported into cryoSPARC (version 4.5.1) for patch-based contrast transfer function (CTF) estimation(7), and 27,934 micrographs with CTF signals extending to 4.5 Å were selected for blob-based automatic particle picking (with particle diameters of 100–160 Å). The blob-picked particles were subjected to 2D classification resulting in a set of 2D averages with structural details expected of the Polε–PCNA particles. These 2D averages were used as templates for the next round of template-based particle picking. A total of 15,815,155 raw particles were automatically picked and 4× binned. After several rounds of 2D classification, particles with clear structural features of the Polε–PCNA complex were selected. In total, 993,543 selected particles were extracted with a box size of 320 pixels and used to compute seven initial 3D models, and selected 3D models with promising structural features were used as 3D template models for heterogenous 3D refinements against all particles. This resulted in seven 3D subclasses. Two low-quality 3D

subclasses reconstructions were discarded as junk. One 3D subclass with good structural features for both Polε and PCNA was selected for homogeneous and non-uniform 3D refinements, resulting in a 3D map at 3.73 Å resolution. The 138,854 particles associated with this 3D map were imported into Relion 5 for Bayesian polishing (10), followed by non-uniform refinement in cryoSPARC, leading to an improved final resolution of 3.60 Å. Two 2D subclasses with the frayed DNA features were combined and subjected to another round of heterogenous refinement. This resulted in a major 3D class with good structural features, which was selected for homogeneous and non-uniform 3D refinements, resulting in a 3D map at 3.64 Å. The 132,717 particles contributing to this map were Bayesian polished in Relion 5 and homogeneous refined in cryoSPARC to produce the final 3.53 Å cryo-EM map. Two remaining 3D subclasses with similar structural features were combined and subjected to a homogenous refinement, resulting in a 3D map at an overall resolution of 3.36 Å. The 3D map has clear density in the Polε region but weaker density in the PCNA region. To address this, a new round of heterogeneous refinement was performed. By discarding a low-resolution 3D subclass and combining the two remaining 3D subclasses for further homogeneous and non-uniform 3D refinements, an improved 3D map at 3.34 Å was obtained. This map was subjected to Bayesian polishing in Relion 5 and non-uniform refinement in cryoSPARC, resulting in the map at 3.11 Å resolution.

Model building, Refinement, and Validation. The cryo-EM structure of human Polε-core–PCNA with an open finger conformation of POLE1-NTD (PDB ID 9B8S (2)) was used as the initial model. The structures of POLE1-NTD and human PCNA were manually docked into the EM maps using UCSF ChimeraX (11). The models were manually adjusted and rebuilt in Coot(8, 12) to fit the EM maps. The original maps were sharpened by EMReady(13) to facilitate modeling of the T/P in the mismatch editing state. The P/T DNA was modified from our published structure in the pol state (PDB ID 9B8T(2)) to fit the T/P EM density in the three proofreading states. Only bases near the mismatched primer 3′-end were rebuilt. Unresolved DNA regions were omitted in all the states. The manually built initial models were subjected to several iterations of real-space refinement in PHENIX (14) and further manual adjustment in Coot (12). All final atomic models were validated using MolProbity (15). The 3D reconstruction and model refinement statistics are provided in **Supplementary Table 1**. Structural figures were prepared in the UCSF ChimeraX(11).

Data Availability. Two cryo-EM 3D maps (3.88 and 3.81 Å) of the ternary human Polε-core–PCNA–T49/P35 (pre-existing mismatch) in the blocked conformations, along with their corresponding atomic models, have been deposited in the Electron Microscopy Data Bank (https://www.ebi.ac.uk/pdbe/emdb/) and the Protein Data Bank (https://www.rcsb.org) under the following accession codes: EMD-49302 and 9NE9 (3.88 Å, The blocked conformation I) and EMD-49303 and 9NEA (3.81 Å, The blocked conformation II). The three 3D EM maps of human Polε-core–PCNA–T47/P29 (with in situ generated mismatch) in three proofreading states (3.60 Å, 3.53 Å, 3.11Å) have been deposited in the EMDB and PDB with accession codes: EMD-49301 and 9NE8 (3.60 Å, Mismatch-locking state), EMD-49300 and 9NE7 (3.53 Å, Pol-backtracking state), and EMD-49299 and 9NE6 (3.11 Å, Mismatch-editing state).

SI Appendix, Video S1. A detailed depiction of the DNA proofreading process by the Polε–PCNA holoenzyme. Morphing of Polε–PCNA proofreading intermediate structures to show the DNA translocation steps and the coordinated conformational changes of Polε.

SI Appendix, Table S1. Cryo-EM data collection, refinement, and validation statistics

	Human polymerase epsilon bound to PCNA and DNA with a pre-existing mismatch in the blocked conformation I (EMD-49302) (PDB 9NE9)	Human polymerase epsilon bound to PCNA and DNA with a pre-existing mismatch in the blocked conformation II (EMD-49303) (PDB 9NEA)	Human polymerase epsilon bound to PCNA and DNA with an in-situ-generated mismatch in the mismatch-locking state (EMD-49301) (PDB 9NE8)	Human polymerase epsilon bound to PCNA and DNA with an in-situ-generated mismatch in the Pol-backtracking state (EMD-49300) (PDB 9NE7)	Human polymerase epsilon bound to PCNA and DNA with an in-situ-generated mismatch in the mismatch-editing state (EMD-49299) (PDB 9NE6)
**Data collection and processing**					
Microscope	FEI Titan Krios		FEI Titan Krios		
Magnification	105,000		105,000		
Voltage (kV)	300		300		
Electron exposure (e^{-}/Å^2)	60		60		
Defocus range (μm)	-1.2 to -1.6		-1.2 to -1.6		
Pixel size (Å/pixel)	0.828		0.828		
Symmetry imposed	C1		C1		
Initial particle images (no.)	978,800		993,543		
Final particle images (no.)	189,127	296,230	138,854	132,717	296,488
Map resolution (Å)	3.88	3.81	3.60	3.53	3.11
FSC threshold	0.143	0.143	0.143	0.143	0.143
Map resolution range (Å)	11.0-3.0	11.0-3.0	8.0-2.0	8.0-2.0	8.0-2.0
**Refinement**					
Model resolution (Å)	4.3	4.3	4.3	4.1	3.7
FSC threshold	0.5	0.5	0.5	0.5	0.5
Model resolution range (Å)	30-3.8	30-3.8	30-3.6	30-3.5	30-3.1
Map sharpening *B* factor (Å^2)	-124.2	-121.3	-131.6	-128.9	-107.2
Model composition					
Non-hydrogen atoms	16735	16578	16242	16138	16349
Protein/DNA residues	1935/65	1936/57	1921/46	1921/41	1923/51
Ligands	3	2	1	1	1
B factors (Å^2)					
Protein/DNA	77.98/155.52	49.15/108.56	116.17/107.65	104.25/167.07	82.05/51.43
Ligand	123.26	20.61	113.25	101.22	68.54
R.m.s. deviations					
Bond lengths (Å)	0.006	0.005	0.005	0.005	0.005
Bond angles (°)	1.213	1.159	1.122	1.110	1.153
Validation					
MolProbity score	1.72	1.84	1.73	1.70	1.56
Clashscore	7.38	8.63	8.88	6.76	6.19
Poor rotamers (%)	0.29	0.12	0.18	0.00	0.00
Ramachandran plot					
Favored (%)	95.43	94.44	96.18	95.29	96.55
Allowed (%)	4.57	5.56	3.82	4.71	3.45
Disallowed (%)	0.00	0.00	0.00	0.00	0.00

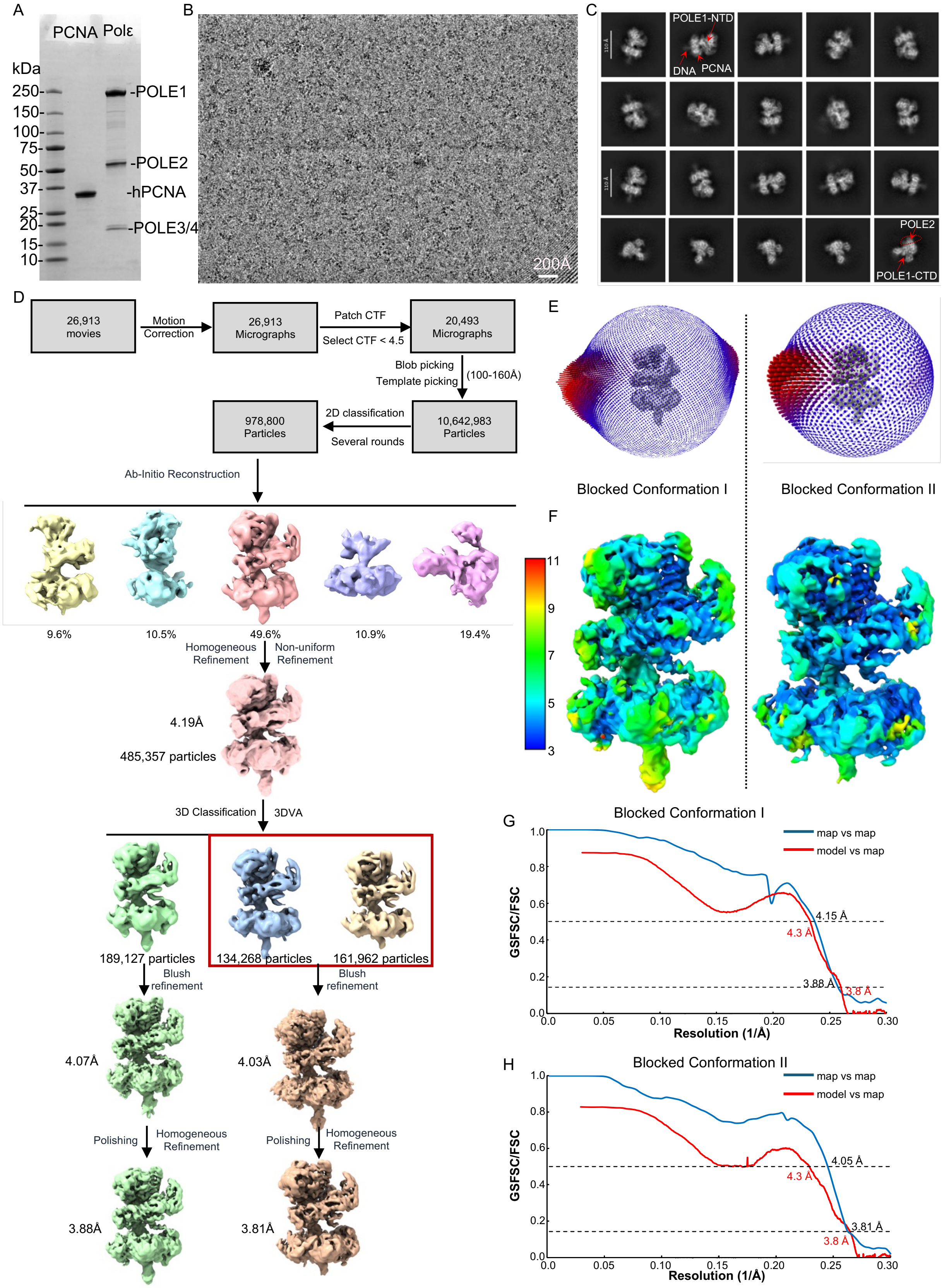

A
PCNA Polε
kDa
250-
150-
100-
75-
50-
37-
25-
20-
15-
10-
-POLE1
-POLE2
-hPCNA
-POLE3/4
B
200Å
C
POLE1-NTD
DNA
PCNA
POLE2
POLE1-CTD
D
26,913 movies
Motion Correction
26,913 Micrographs
Patch CTF
Select CTF < 4.5
20,493 Micrographs
Blob picking
Template picking
(100-160Å)
978,800 Particles
2D classification
Several rounds
10,642,983 Particles
Ab-Initio Reconstruction
9.6%
10.5%
49.6%
10.9%
19.4%
Homogeneous Refinement
Non-uniform Refinement
4.19Å
485,357 particles
3D Classification
3DVA
189,127 particles
134,268 particles
161,962 particles
Blush refinement
Blush refinement
4.07Å
4.03Å
Polishing
Homogeneous Refinement
Polishing
Homogeneous Refinement
3.88Å
3.81Å
E
Blocked Conformation I
Blocked Conformation II
F
11
9
7
5
3
G
Blocked Conformation I
map vs map
model vs map
GSFSC/FSC
4.15 Å
4.3 Å
3.88 Å
3.8 Å
Resolution (1/Å)
H
Blocked Conformation II
map vs map
model vs map
GSFSC/FSC
4.05 Å
4.3 Å
3.81 Å
3.8 Å
Resolution (1/Å)

SI Appendix, Fig. S1. Cryo-EM data processing of the Polε–PCNA–T49/P35 ternary complex (with pre-existing mismatch). (A) SDS-PAGE gel of purified wild-type human Polε and PCNA. (B) Typical raw micrograph of the in vitro assembled complex. A total of 26,913 micrographs were recorded. (C) Selected 2D class averages in various views. (D) Workflow of cryo-EM data processing and 3D reconstruction in CryoSPARC (version 4.5.1), leading to the two 3D EM maps at 4.01 Å and 3.95 Å, respectively. (E) Angular distribution of particle images contributing to the two final 3D reconstructions. (F) Color-coded local resolution maps of the ternary complex in two blocked conformations. (G-H) Gold standard Fourier shell correlation (GSFSC) of the two EM maps (blue) and the model-to-EM map correlation curve (red) of the ternary complex in two blocked conformations.

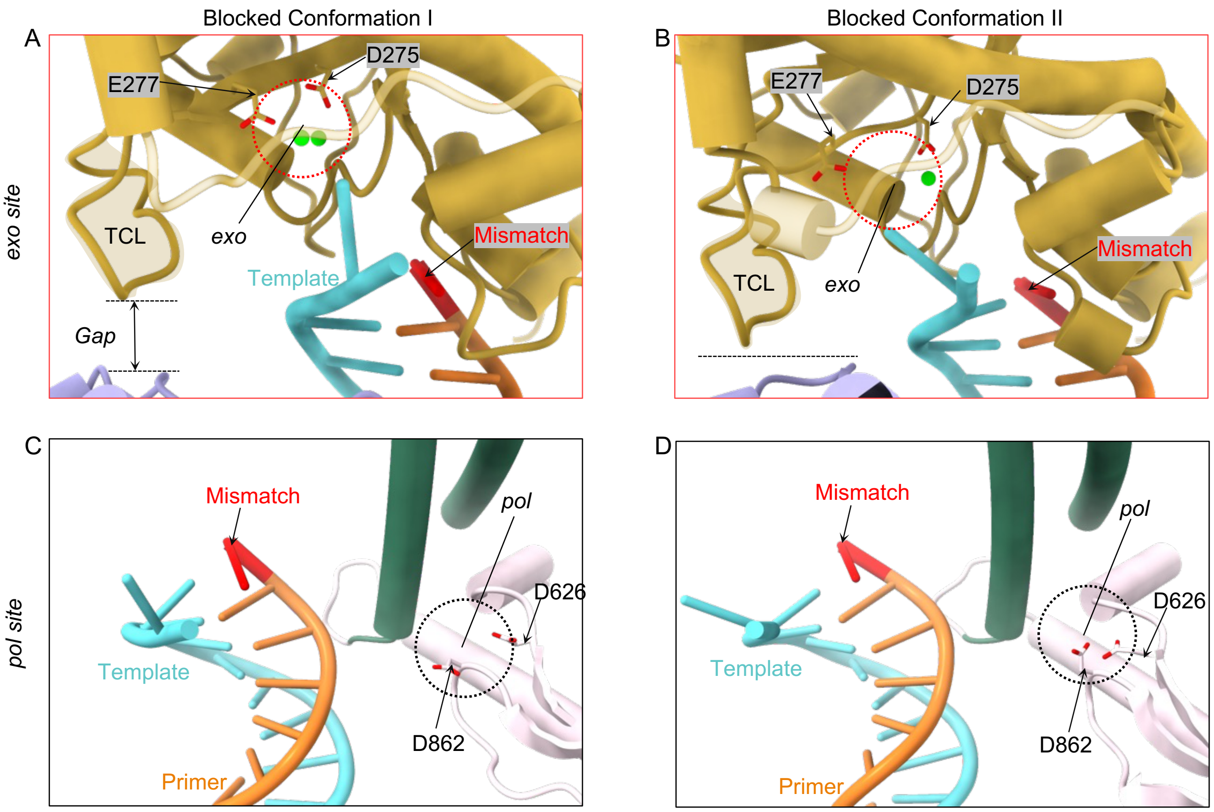

SI Appendix, Fig. S2. DNA positions in the Polε–PCNA–T49/P35 (with pre-existing mismatch) in the two blocked conformations. (A-B) The mismatched primer 3′-end (red stick) with respect to the Polε *exo* site (dashed red circle) of the holoenzyme in blocked conformations I (A) and II (B). The mismatched primer 3′-end is far away from the *exo* site. There is a gap between the exo and thumb domains in the blocked conformation I, but the gap is closed via a TCL loop in the blocked conformation II. The gap may facilitate the template strand to enter a channel leading up to the *exo* site. (C-D) The mismatched 3′-end (red stick) with respect to the *pol* site (dashed black circle) in blocked conformation I (C) and conformation II (D). The pol site contains no catalytic metal ions to coordinate the catalytic residues D626 and D862 residues, and the mismatched 3′-end is far from the *pol* site.

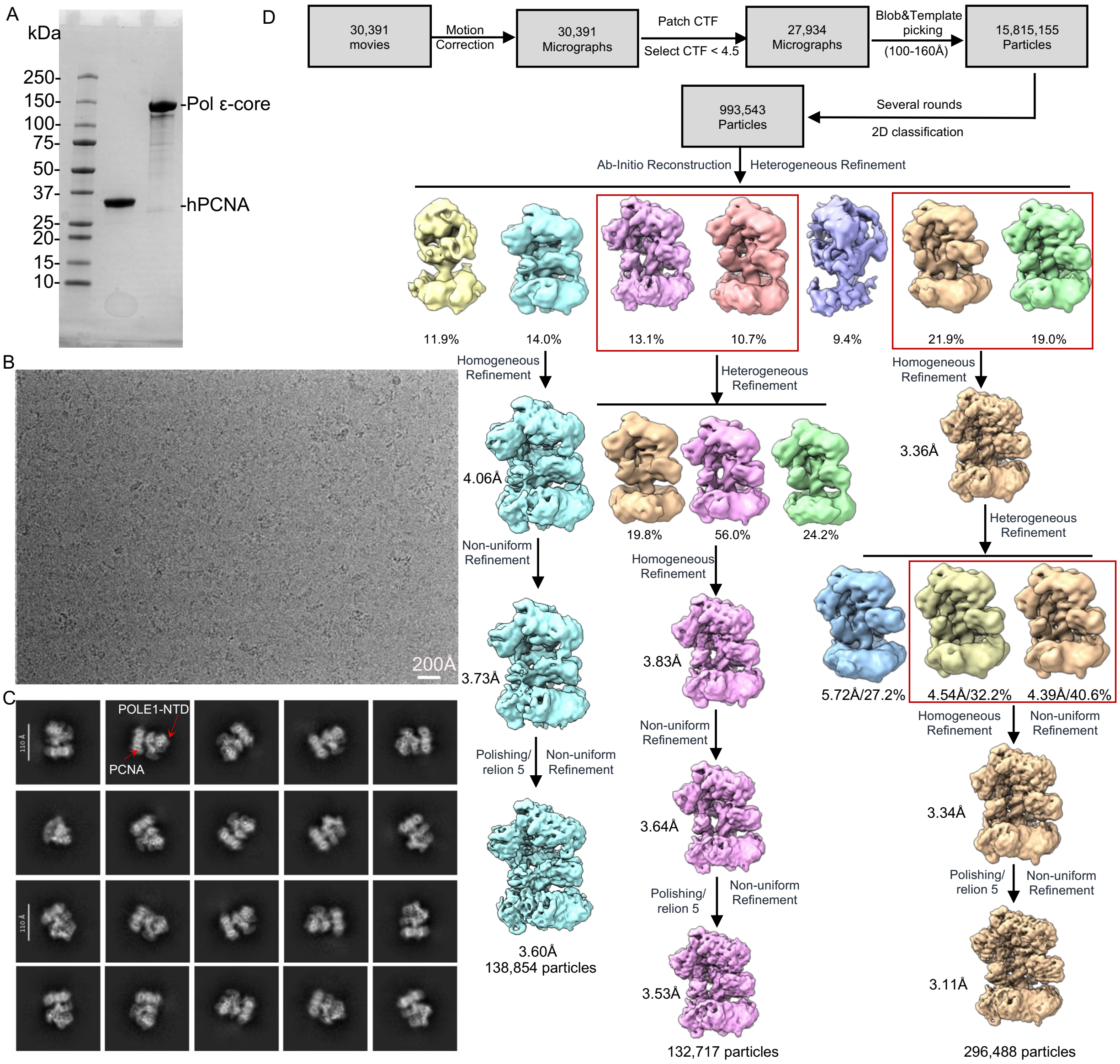

SI Appendix, Fig. S3. Cryo-EM data processing of the Polε–PCNA–T47/P29 (with in-situ-generated mismatch). (A) SDS-PAGE gel of purified human Polε-core exo^{-} and PCNA. (B) Typical raw micrograph out of the total 30,391 recorded movies. (C) Selected 2D class averages in various views. (D) Workflow of cryo-EM data processing and 3D reconstruction in CryoSPARC (version 4.5.1), leading to three 3D EM maps at 3.60 Å, 3.53 Å, and 3.11 Å average resolution, respectively. The final Bayesian polishing was performed by Relion-5.

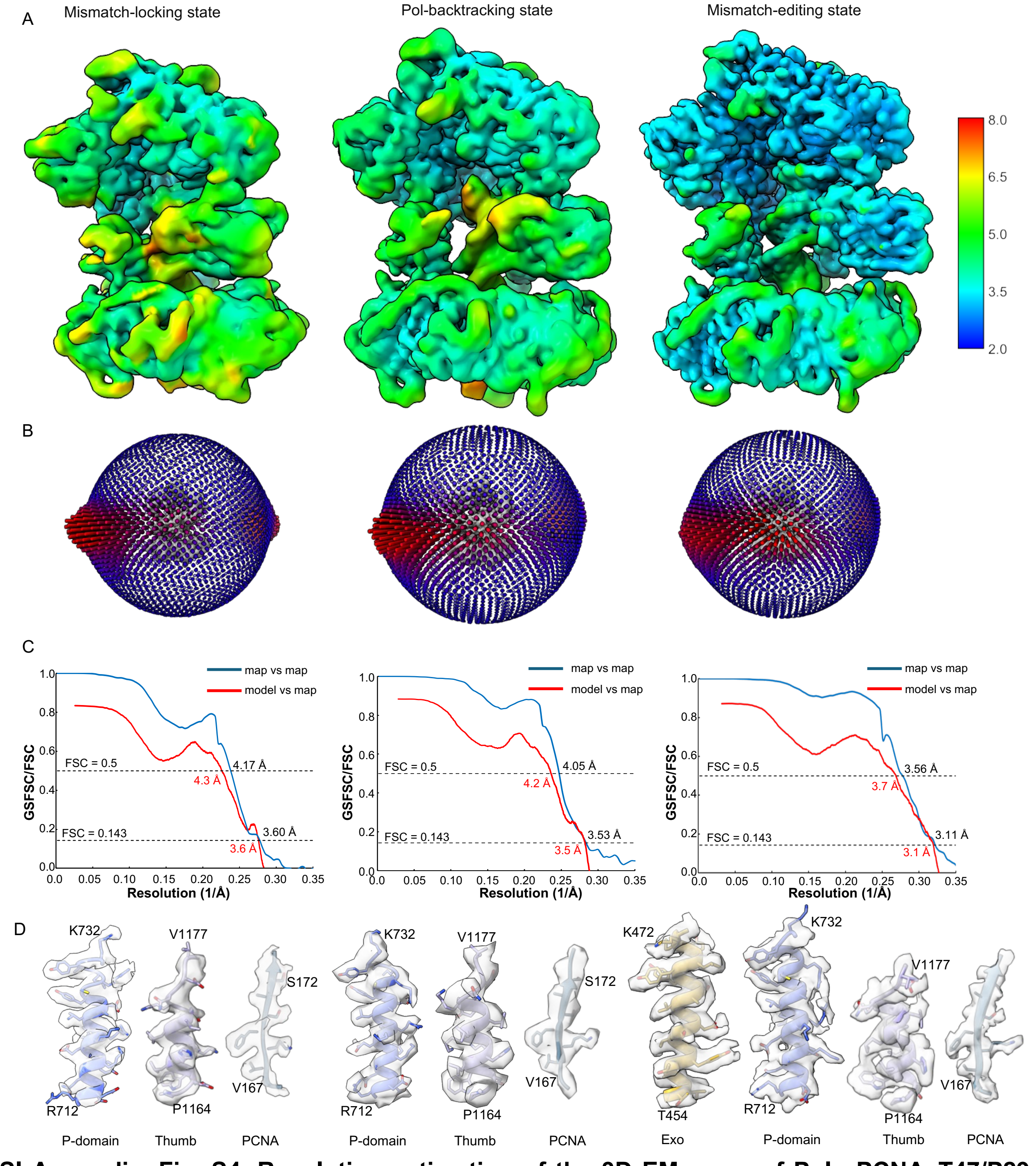

SI Appendix, Fig. S4. Resolution estimation of the 3D EM maps of Polε–PCNA–T47/P29 (with in-situ-generated mismatch) in three proofreading states. (A) Color-coded local resolution maps of three proofreading states. (B) Angular distribution of particle images used in the final 3D reconstructions. (C) Gold standard Fourier shell correlation (GSFSC, blue) and the map-to-model correction (red) curves. (D) EM densities in three selected regions of each of the three proofreading states.

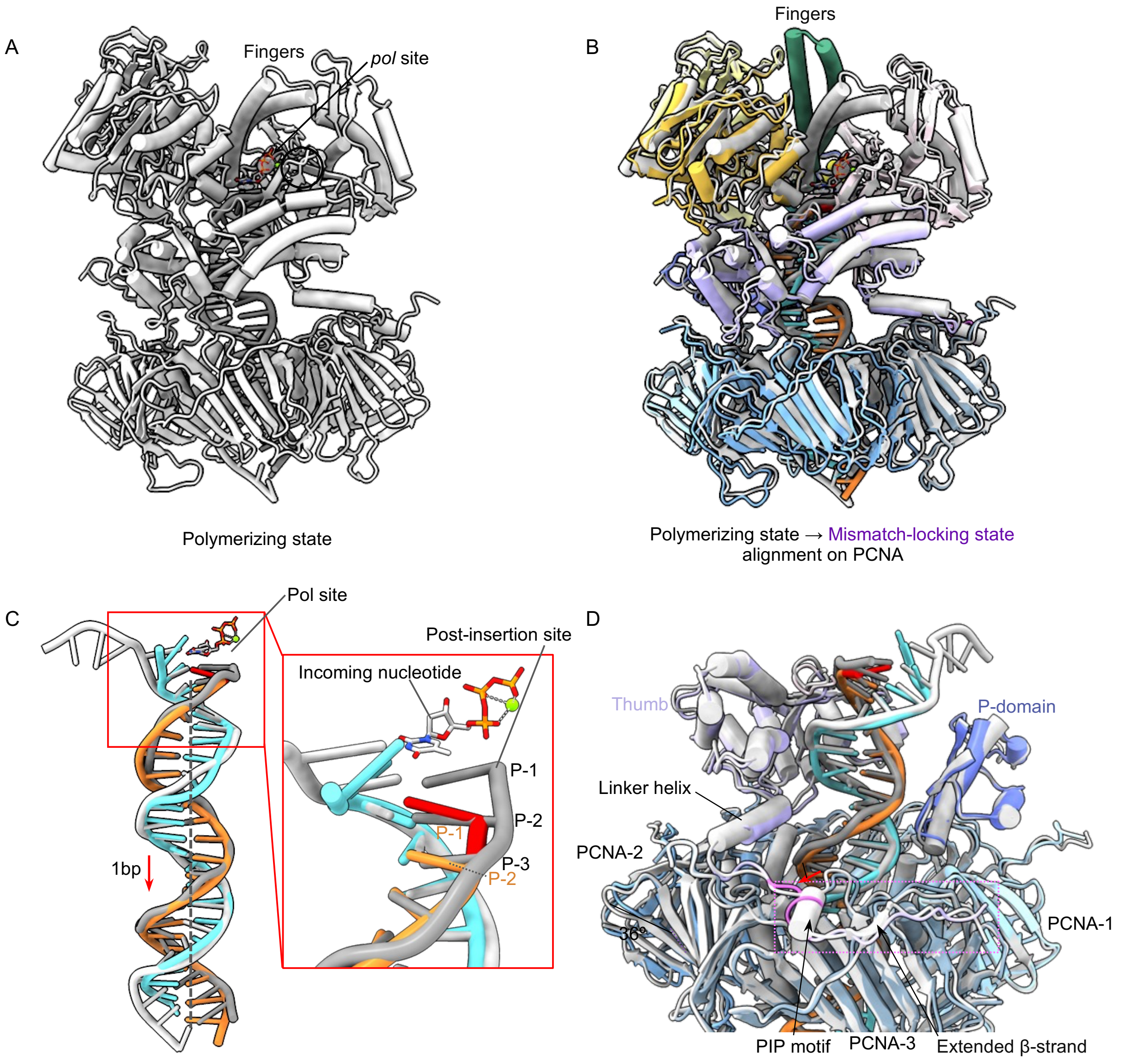

SI Appendix, Fig. S5. Conformational changes of the human Polε holoenzyme from the polymerization to the mismatch-locking state. (A) Structure of the reported polymerization state (PDB ID 9B8T) of Polε–PCNA with a matched T/P. (B) Superimposition of the polymerization (gray) and mismatch-locking (color) states reveals that the Fingers domain transitions from the closed (gray) to open (dark green) conformation. (C) Superposition of the T/P reveals a 1-bp downward shift from the polymerization to the mismatch-locking state. (D) No major changes occur at the Polε-PCNA interface region from the polymerization (gray) to the mismatch-locking (color) states.

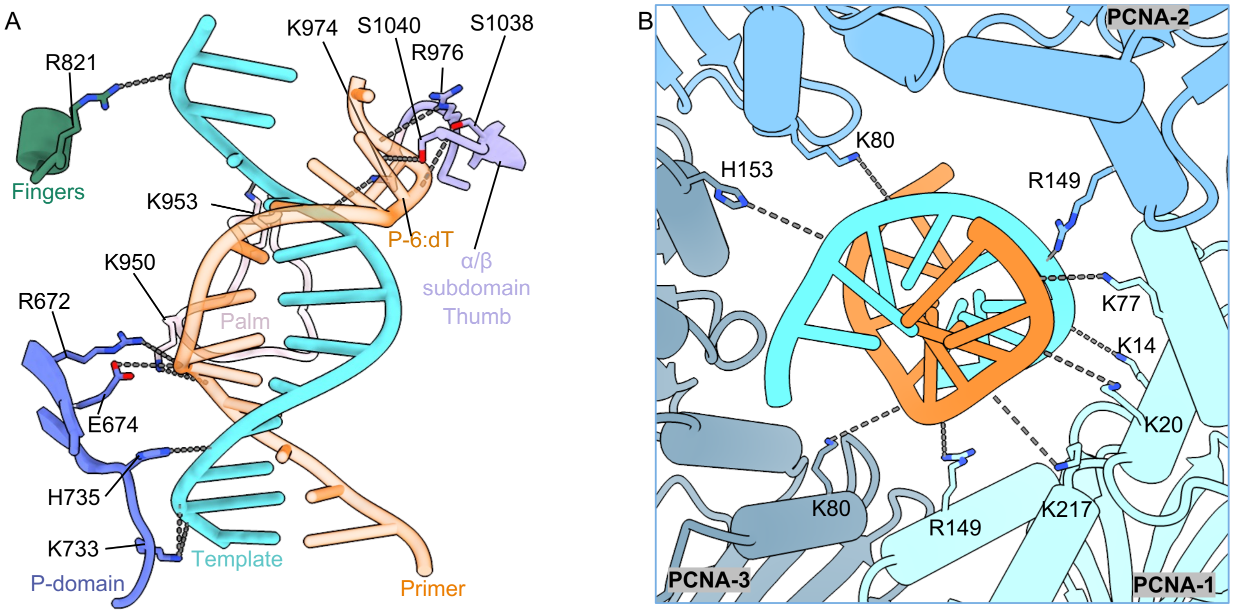

SI Appendix, Fig. S6. The duplex region of the T/P is well stabilized by the Polε–PCNA holoenzyme during mismatch-editing. (A) The T/P duplex region above PCNA interacts with residues from the Fingers, P-domain, and thumb domain. (B) The T/P duplex region inside PCNA is slightly tilted and interacts with 9 positively charged residues.

References for SI Appendix

1. F. Weissmann *et al.*, biGBac enables rapid gene assembly for the expression of large multisubunit protein complexes. *Proc. Natl. Acad. Sci. U.S.A.* **113**, E2564-E2569 (2016).
2. Q. He, F. Wang, N. Y. Yao, M. E. O'Donnell, H. Li, Structures of the human leading strand Polε–PCNA holoenzyme. *Nat. Commun.* **15**, 7847 (2024).
3. F. Wang, Q. He, N. Y. Yao, M. E. O'Donnell, H. Li, The human ATAD5 has evolved unique structural elements to function exclusively as a PCNA unloader. *Nat. Struct. Mol. Biol.* **31**, 1680–1691 (2024).
4. J. J. Roske, J. T. P. Yeeles, Structural basis for processive daughter-strand synthesis and proofreading by the human leading-strand DNA polymerase Pol ε. *Nat. Struct. Mol. Biol.* **31**, 1921–1931 (2024)
5. D. N. Mastronarde, Automated electron microscope tomography using robust prediction of specimen movements. *J. Struct. Biol.* **152**, 36-51 (2005).
6. S. Q. Zheng *et al.*, MotionCor2: anisotropic correction of beam-induced motion for improved cryo-electron microscopy. *Nat. methods* **14**, 331-332 (2017).
7. A. Punjani, J. L. Rubinstein, D. J. Fleet, M. A. Brubaker, cryoSPARC: algorithms for rapid unsupervised cryo-EM structure determination. *Nat. methods* **14**, 290-296 (2017).
8. A. Punjani, D. J. Fleet, 3D variability analysis: Resolving continuous flexibility and discrete heterogeneity from single particle cryo-EM. *J. Struct. Biol.* **213**, 107702 (2021).
9. D. Kimanius *et al.*, Data-driven regularization lowers the size barrier of cryo-EM structure determination. *Nat. Methods* **21**, 1216-1221 (2024).
10. A. Burt *et al.*, An image processing pipeline for electron cryo-tomography in RELION-5. *FEBS Open Bio* (2024).
11. E. F. Pettersen *et al.*, UCSF ChimeraX: Structure visualization for researchers, educators, and developers. *Protein Sci.* **30**, 70-82 (2021).
12. P. Emsley, B. Lohkamp, W. G. Scott, K. Cowtan, Features and development of Coot. *Acta Crystallogr. D* **66**, 486-501 (2010).
13. J. He, T. Li, S.-Y. Huang, Improvement of cryo-EM maps by simultaneous local and non-local deep learning. *Nat. Commun.* **14**, 3217 (2023).
14. P. V. Afonine *et al.*, Real-space refinement in PHENIX for cryo-EM and crystallography. *Acta Crystallogr. D* **74**, 531-544 (2018).
15. C. J. Williams *et al.*, MolProbity: More and better reference data for improved all-atom structure validation. *Protein Sci.* **27**, 293-315 (2018).
